# Supplementary material for: GO Bench: shared hub for universal benchmarking of machine learning-based protein functional annotations
Source: Bioinformatics. 2023 Feb 14;39(2):btad081. doi: 10.1093/bioinformatics/btad081 (PMC10132473; doi:10.1093/bioinformatics/btad081)
Supplement: btad081_Supplementary_Data [file btad081_supplementary_data.pdf]

Bioinformatics
doi.10.1093/bioinformatics/btad081
Advance Access Publication Date: 14 Feb 2023
Application Note

Sequence Analysis

GO Bench: Shared-hub for Universal Benchmarking of Machine Learning-Based Protein Functional Annotations

Andrew Dickson*[1], Ehsaneddin Asgari*[1,2], Alice C. McHardy[2], and Mohammad R.K. Mofrad[1†]

[1]Molecular Cell Biomechanics Laboratory, Departments of Bioengineering and Mechanical Engineering, University of California, Berkeley, CA, 94720, USA
[2]Computational Biology of Infection Research, Helmholtz Centre for Infection Research, Brunswick 38124, Germany

* Equal contribution.
† To whom correspondence should be addressed.

Abstract

Contact: amdickson@berkeley.edu
Supplementary information: Supplementary information contained here.

1 Data Preparation

Annotation data and the gene ontology itself are downloaded from the Gene Ontology Association (GOA) database and processed with in-house scripts developed in Python. Code is included in https://github.com/mofradlab/go_bench. For model training, protein identifiers and sequences are downloaded separately from UniProt's SwissProt database.

The GOA database is the April 2021 version from "ftp://ftp.ebi.ac.uk". It takes the form of a tab separated CSV file, with fields for UniProt protein ID, Gene Ontology annotation, date of annotation, and annotation qualifiers such as "NOT", "contributes to", or "colocalizes with". Each row of the GOA database represents a single annotation for a specific protein.

The entire dataset is prefiltered to removed annotations not for Swissprot proteins. Any annotations with negative qualifiers are also removed. All GO Bench datasets are sourced from a local version of the filtered GOA dataset. For model training, and to filter non-Swissprot proteins, we use the August 2021 version of Uniprot, located at "ftp.uniprot.org/pub/databases/uniprot/previous_major_releases".

2 Train-Test Split

We provide two train-validation-test splits for user downloads, named cluster50 and random.

All sequences used for our cluster50 datasets are extracted from the UniRef50 dataset (SwissProt), in which sequences are clustered to 50% identity. Clusters are randomly assigned to one of training, validation, or testing categories until they are as close as possible to fractions of 70, 15, and 15 percent respectively. This guarantees that proteins with over 50% similarity are never assigned to different portions of the dataset, which would compromise the integrity of several validation functions. Our random dataset is primarily for reference, and is generated using the same proteins, but without accounting for clustering. Splits are entirely random, but have the same proportions.

Once users select their train-test split, datasets may be further customized with operations such as annotation propagation, filtering, or the addition of estimated negative annotations. However, regardless of customizations, the underlying train-test split remains the same, so that the resulting models can be safely added to the corresponding leaderboard.

2.1 Cluster50 Stability

A possible issue with splitting training data by large 50% similarity clusters is high variance in training and testing set statistics. To evaluate the impact of selecting a cluster50 train-validation-test split, we randomly and independently generate five splits with methodology identical to that described above. Within each set, we only consider the gene ontology terms associated with over 50 proteins as suitable for training, so the precise gene ontology terms selected for a given train-test split depends slightly on the split distribution. If there were excessive differences between random splits, it could indicate that model results on the single split used in our webserver would be too random, so we compare the overall results for a generic model in each dataset. For each random split, we train the same DGP Convolutional model, and compare the resulting testing performance metrics. As shown below, for each of our three testing metrics, there's

© The Author 2023. Published by Oxford University Press. All rights reserved. For permissions, please e-mail: journals.permissions@oup.com

low variance in results, with the largest difference being for the S_{min} metric, in which large relative differences between GO term weightings may introduce additional variance.

Split	Molecular Function		
	F_{max}	S_{min}	F_{score}
Split 0	0.696	21.4	323
Split 1	0.696	18.7	316
Split 2	0.694	21.5	318
Split 3	0.695	22.1	321
Split 4	0.699	22.2	328
Aggr	0.696 ±0.00167	21.2 ±1.26	321.28 ±3.90

3 Model Architecture

Benchmark models were developed using Pytorch, the BLAST package, and BLAST annotation scripts provided by CAFA. All code is included in the on github at github.com/mofradlab/GO_benchmarking.

BLAST annotations were generated using the same process and methodology as in CAFA, but with our own training data.

For our deep learning benchmarks, we chose several basic models, a feedforward neural network taking in a 3-mer embedding, an LSTM taking in sequences directly, and a convolutional model also taking in sequences directly.

Our 3-mer embedding was generated by feeding our training dataset into the tf-idf transform from the sklearn python library. The transform extracts the count of every possible sequential triple of amino acids in a sequence, and then downweights by a heuristic measure to prevent the most common triples from dominating. This was then combined with the mean of ProtVec embeddings of all 3-mers in a protein to generate a composite vector of roughly 10,000 entries.

The feedforward network is implemented in Pytorch. It contains one input layer, two hidden layers of size 5000, and one output layer generating probabilities for each GO term in parallel. Between each layer, we used Dropout with rate 0.5, BatchNorm regularizers and ReLU non-linearities. For each GO domain, we use the same network architecture, and a network is trained for each domain.

Our convolutional network is built implemented in Pytorch, using methodology from the network component of the Deep Pred GO model. It is a shallow convolutional model which applies 1D convolutional filters ranging in size from 3 to 80 the entire protein sequence, and then applies a fully-connected feedforward layer to their concatenated output. Full details of the convolutional implementation are included in the DeepGoPlus paper, as well as our codebase.

4 Model Training

Deep learning models are trained using the Pytorch Lightning package. Each uses Lightning's heuristically determined learning rate on a linearly decaying schedule, and the Adam optimizer for gradient descent. For all networks, we use a batch size of 128 All networks are trained on a GeForce 1080 GPU until validation performance decreases for three successive epochs.

5 Performance Metrics

GO Bench uses the F-max, S-min, and F-score metrics mentioned previously. The F-max and S-min metrics are directly based off of the CAFA competition, and are described in detail in the CAFA 2 paper "An expanded evaluation of protein function prediction methods shows an improvement in accuracy" (Jiang and Oron, 2016). The 'F-Score' metric is defined in "Learning from Positive and Unlabeled Data: a survey" (Bekker and Davis, 2020). For clarification, we describe all metrics below.

Let G_n represent the set of GO terms in a given namespace n, and P represent the set of proteins within our dataset. For the purpose of metric evaluation, we write GO annotation as a binary classification problem, in which a pair $x = (g \in G_n, p \in P)$ is classified as either true or false.

Define classification models as functions parameterized by θ mapping GO term, protein pairs to probability estimates. $M_\theta|G_n \times P \to [0, 1]$. At a given threshold α, the classification estimate for an input $x = (g, p)$ is $\hat{y} = M_\theta(x) \geq \alpha$.

GO Bench datasets consist of positive GO annotation for a a given set of proteins (within the constraints of customization). Negative annotation is represented implicitly by absence from the dataset. We write the complete dataset as $D = (X, y)$, where X represents all pairs of proteins included in the dataset and GO terms, and $y \in \{0, 1\}$ gives the classification label for that pair. Assume there are N datapoints, with (x_i, y_i) giving the ith pair.

5.1 F-Max

For a given α, define precision as the number of true positive predictions divided by the number of positive predictions and recall as the number of true positive predictions divided by the number positive annotations.

$$pr(\alpha) = \frac{\sum_{i=1}^{N} 1(y_i = \hat{y}_i(\alpha))}{\sum_{i=1}^{N} \hat{y}_i(\alpha)} \quad (1) \qquad rc(\alpha) = \frac{\sum_{i=1}^{N} 1(y_i = \hat{y}_i(\alpha))}{\sum_{i=1}^{N} y_i} \quad (2)$$

The F1 score is defined as $\frac{2pr(\alpha)rc(\alpha)}{pr(\alpha)+rc(\alpha)}$, and the F-max score is $\max_{\alpha\in[0,1]} F1(\alpha)$. When calculating the F1 score, we use the 'micro-F1' score defined in the python sklearn package (Pedregosa *et al.*, 2011), and evaluate results over threshold values ranging from 0 to 1 over increments of 0.01 to take the maximum.

5.2 S-min

Information content is a quantitative measure of the descriptive value of a gene ontology term, defined using the GO graph structure (Clark and Radivojac, 2013). It associates a value $ic(g)$ to each GO term $g \in G_n$.

With information content, define remaining uncertainty and missing information as the following.

$$ru(\alpha) = \frac{1}{|P|} \sum_{i=1}^{N} ic(g_i) \cdot 1(\hat{y}_i(\alpha) = 0 \wedge y_i = 1) \quad (3)$$

$$mi(\alpha) = \frac{1}{|P|} \sum_{i=1}^{N} ic(g_i) \cdot 1(\hat{y}_i(\alpha) = 1 \wedge y_i = 0) \quad (4)$$

The final S-min score is $S_{min} = min_\alpha \left(\sqrt{ru(\alpha)^2 + mi(\alpha)^2}\right)$.

5.3 F-Score

The F-Score metric is defined to approximate the true F1 score with perfect knowledge of datapoint labels (Bekker and Davis, 2020). It's based on recall, $rc(\alpha)$, and the probability of a positive prediction, $P(\hat{y}(\alpha) = 1) = \frac{1}{N} \sum_{i=1}^{N} \hat{y}(\alpha)$. For a given α, the F-Score is

$$\frac{rc(\alpha)^2}{P(\hat{y}(\alpha) = 1)}$$

As with F-max, we take the maximum over all thresholds α for our results.

6 Website

The GO Bench website is hosted at www.gobench.org. It contains a simple webform for customizing and downloading datasets, visualizations of class frequencies within downloaded datasets, a leaderboard for submitted models, and scoring visualizations for uploaded models.

7 Codebase

GOBench and its associated utilities are developed in Python 3.7, using the Numpy, Pandas, Sklearn, Scipy, and GOATools scientific packages (Harris *et al.*, 2020; Buitinck *et al.*, 2013; Virtanen *et al.*, 2020; Klopfenstein *et al.*, 2018; pandas development team, 2020).

7.1 GOBench Package

Although our benchmark code is primarily for use in the web-application, it may be useful for rapidly generating custom datasets, and parsing Gene Ontology data. Non-specific utilities from our application are packaged in the gobench git repository at github.com/mofradlab/go_bench. It contains functions for directly converting raw datasets of Gene Ontology Annotations into GOBench formatted datasets, including a pipeline function with configurable settings and file inputs, and utility functions for reading and writing gene ontology files.

Usage of the GOBench package, including download links for required datasets and pre-generated train-test splits, is shown in IPython notebooks contained within the repository. The train-test split included in the repository by default is identical to the one used in our web-application, and can be used to replicate any of our results.

The gobench package is configured to be pip installable. It can be installed directly from git using instructions in the repository, and used directly to reproduce or expand upon our application.

7.2 Example Code

In combination, our web-application and gobench python package make it possible to quickly generate and use Gene Ontology datasets for machine learning training and benchmarking. As a simple example of usage, we include an annotated sample repository containing the complete process of downloading a GOBench dataset, using it to train and evaluate a model, and uploading predictions to the GOBench leaderboard. All code and date is stored in another git repository at github.com/mofradlab/GO_Bench_Sample. The notebook can also be run in browser from a Google Colab notebook.

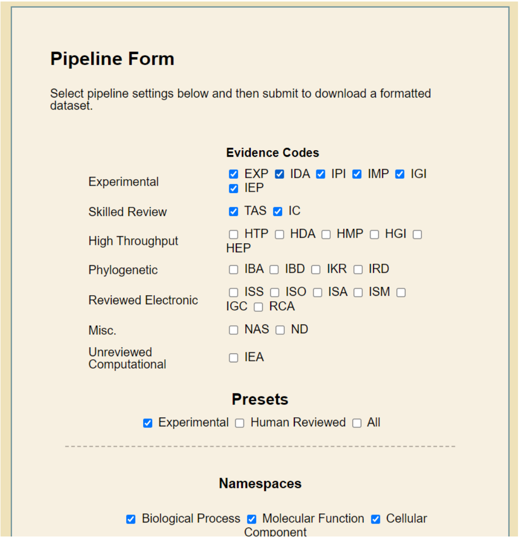

Fig. 1: Initial form for generating images. Form is built from the HTML <form> element, and contains entries for annotation quality, namespaces, annotation propagation, term filtration, train-test split method, and cutoff dates for annotation assignment.

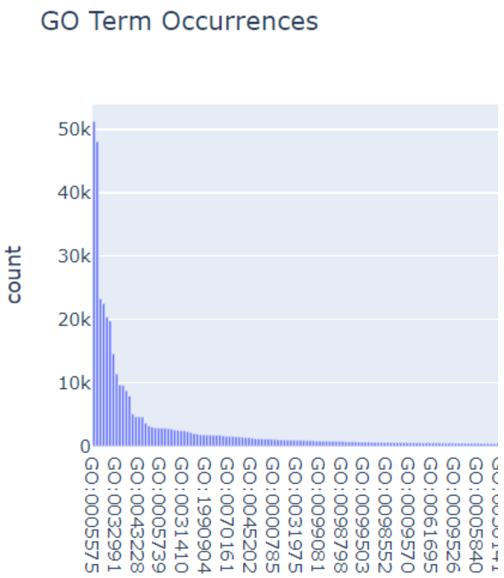

Fig. 2: Histogram visualization of GO class counts for each GO entry. Separate counts are given for each namespace in the gene ontology (Molecular Function, Cellular Component, and Biological Process). Common terms such as protein interaction appear in tens of thousands of dataset proteins, while the rarest ones appear only once, and are usually filtered out by form settings.

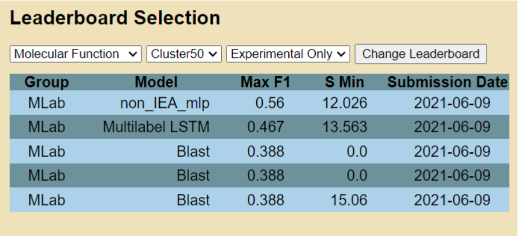

Leaderboard Selection

Molecular Function | Cluster50 | Experimental Only | Change Leaderboard

Group	Model	Max F1	S Min	Submission Date
MLab	non_IEA_mlp	0.56	12.026	2021-06-09
MLab	Multilabel LSTM	0.467	13.563	2021-06-09
MLab	Blast	0.388	0.0	2021-06-09
MLab	Blast	0.388	0.0	2021-06-09
MLab	Blast	0.388	15.06	2021-06-09

Fig. 3: The GO Bench leaderboard records F-Max, S-Min, and F-Score metrics for each uploaded model, and presents a top ten list for the highest ranking versions.

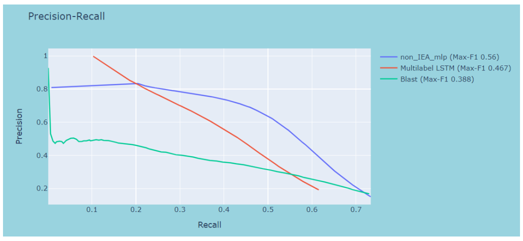

Fig. 4: The GO Bench leaderboard is also be visualized using plots of scoring metrics including precision-recall.

References

Bekker, J. and Davis, J. (2020). Learning from positive and unlabeled data: a survey. *Machine Learning*, **109**(4), 719–760.

Buitinck, L., Louppe, G., Blondel, M., Pedregosa, F., Mueller, A., Grisel, O., Niculae, V., Prettenhofer, P., Gramfort, A., Grobler, J., Layton, R., VanderPlas, J., Joly, A., Holt, B., and Varoquaux, G. (2013). API design for machine learning software: experiences from the scikit-learn project. In *ECML PKDD Workshop: Languages for Data Mining and Machine Learning*, pages 108–122.

Clark, W. T. and Radivojac, P. (2013). Information-theoretic evaluation of predicted ontological annotations. *Bioinformatics*, **29**(13), i53–i61.

Harris, C. R., Millman, K. J., van der Walt, S. J., Gommers, R., Virtanen, P., Cournapeau, D., Wieser, E., Taylor, J., Berg, S., Smith, N. J., Kern, R., Picus, M., Hoyer, S., van Kerkwijk, M. H., Brett, M., Haldane, A., del Río, J. F., Wiebe, M., Peterson, P., Gérard-Marchant, P., Sheppard, K., Reddy, T., Weckesser, W., Abbasi, H., Gohlke, C., and Oliphant, T. E. (2020). Array programming with NumPy. *Nature*, **585**(7825), 357–362.

Jiang, Y. and Oron, T. R. e. a. (2016). An expanded evaluation of protein function prediction methods shows an improvement in accuracy. *Genome Biology*, **17**(1), 184.

Klopfenstein, D. V., Zhang, L., Pedersen, B. S., Ramírez, F., Warwick Vesztrocy, A., Naldi, A., Mungall, C. J., Yunes, J. M., Botvinnik, O., Weigel, M., Dampier, W., Dessimoz, C., Flick, P., and Tang, H. (2018). Goatools: A python library for gene ontology analyses. *Scientific Reports*, **8**(1), 10872.

pandas development team, T. (2020). pandas-dev/pandas: Pandas.

Pedregosa, F., Varoquaux, G., and et. al. (2011). Scikit-learn: Machine learning in Python. *Journal of Machine Learning Research*, **12**, 2825–2830.

Virtanen, P., Gommers, R., Oliphant, T. E., Haberland, M., Reddy, T., Cournapeau, D., Burovski, E., Peterson, P., Weckesser, W., Bright, J., van der Walt, S. J., Brett, M., Wilson, J., Millman, K. J., Mayorov, N., Nelson, A. R. J., Jones, E., Kern, R., Larson, E., Carey, C. J., Polat, İ., Feng, Y., Moore, E. W., VanderPlas, J., Laxalde, D., Perktold, J., Cimrman, R., Henriksen, I., Quintero, E. A., Harris, C. R., Archibald, A. M., Ribeiro, A. H., Pedregosa, F., van Mulbregt, P., and SciPy 1.0 Contributors (2020). SciPy 1.0: Fundamental Algorithms for Scientific Computing in Python. *Nature Methods*, **17**, 261–272.
